# Influence of free charge carrier density on the magnetic behavior of (Zn,Co)O thin film studied by Field Effect modulation of magnetotransport

**DOI:** 10.1038/s41598-018-36336-w

**Published:** 2019-01-16

**Authors:** E. Bellingeri, S. Rusponi, A. Lehnert, H. Brune, F. Nolting, A. Leveratto, A. Plaza, D. Marré

**Affiliations:** 1 CNR-SPIN C.so F. M. Perrone, 24, 16152 Genova, Italy; 2 Dipartimento di Fisica, Università di Genova, Via Dodecaneso 33, 16146 Genova, Italy; 3 Ecole Polytechnique Fédérale de Lausanne (EPFL), CH-1015 Lausanne, Switzerland; 4 Swiss Light Source, Paul Scherrer Institut, CH-5232 Villigen PSI, Switzerland

## Abstract

The origin of (ferro)magnetic ordering in transition metal doped ZnO is a still open question. For applications it is fundamental to establish if it arises from magnetically ordered impurity clusters embedded into the semiconducting matrix or if it originates from ordering of magnetic ions dilute into the host lattice. In this latter case, a reciprocal effect of the magnetic exchange on the charge carriers is expected, offering many possibilities for spintronics applications. In this paper we report on the relationship between magnetic properties and free charge density investigated by using Zinc oxide based field effect transistors, in which the charge carrier density is modulated by more than 4 order of magnitude, from 10^16^ to 10^20^ e^−^/cm^3^. The magnetotransport properties are employed to probe the magnetic status of the channel both in pure and cobalt doped zinc oxide transistors. We find that it is widely possible to control the magnetic scattering rates by field effect. We believe that this finding is a consequence of the modulation of magnetization and carrier spin polarization by the electric field. The observed effects can be explained by the change in size of bound magnetic polarons that induces a percolation magnetic ordering in the sample.

## Introduction

Dilute magnetic semiconductors (DMS), in which a fraction of atoms of the nonmagnetic semiconductor host is replaced by magnetic ions, have recently attracted broad interest for their potential application in spintronics. Ferromagnetism has been reported in III-V and II-IV semiconductors doped with magnetic ions; however, their transition temperatures are far too low for possible room temperature (RT) applications. Transition metal (TM) doped Zinc oxide seems a good alternative because RT ferromagnetism was both theoretically predicted^1^ and experimentally observed^2–7^. However, the origin of such ferromagnetism and in particular if it is a signature of a true DMS behaviour (long range magnetic interaction between the doping ions) or if it arises from the formation of secondary phases, segregation or clustering^8–11^ is still under debate^12^.

In this respect, measuring the dependence of the magnetic properties on the carrier concentration can actually clarify the underlying physics and establish if the TM doped ZnO is a DMS. This is usually done by comparing the magnetic properties of samples with identical TM doping but different carrier concentration modulated by adding a second doping element, for example Aluminium^3,13^. In such samples, varying by a few per cent the Al doping level, changes from 0 to 4 µ_B_ the magnetic moment of the TM ions^13^.However, such experiments suffer the limitation of comparing samples with different crystallographic structure and different disorder generated by the different doping level. As pointed out in the excellent reviews on this subject by S. Ogale^14^ and Z. Yang^15^ there is the necessity to perform specific experiment based on the modulation of the carrier density by Field Effect (FE) in these materials and to focus the attention not only on the magnetization but also on the often neglected carrier spin polarization, a key property in view of spintronics application. FE experiments have the evident advantage of measurements performed on the same sample; consequently, the magnetic moment of the TM ions can be modulated by simply varying the gate voltage, without any change in the magnetic ion distribution and in the crystallographic environment. So far, only a few studies have used electrostatic doping to investigate the effect of carrier concentration on the magnetic properties of thin Co doped ZnO films^16,17^, and Mn doped ZnO nanowires^18^. However, in all these cases, FE was only able to induce very small changes, of the order of a few percent, in the carrier concentration. In other cases, electric control of magnetism in ZnO compounds is not related to pure FE but to drift and/or formation of oxygen vacancy resulting in a non-volatile effect^19^. Recently, it has also been reported that magnetism in ZnO films could not only depend on the oxygen vacancy concentration but also on the effect of UV light on the distribution of their ionization states^20^.

In order to realize a large charge modulation we have grown Co-doped ZnO/Strontium Titanate (STO) heterostructures^21^. By exploiting the high-k properties of STO, whose dielectric constant ε_r_ raises up to 10000 at low temperature (compared with ε_r_ ∼ 5 for SiO_2_), we are able to fabricate FE devices in which the carrier density can be modulated by more than four orders of magnitude^22–24^. Such devices, realized in side gate geometry, allow a direct access to the transport channel, and are thus suitable for the study of the intrinsic properties of the material, and in particular of their dependence on the free carrier concentration^25^. By performing magnetoresistance experiments under FE, we observed a clear correlation between carrier concentration and magnetism. In particular our data show that TM doped ZnO is not a “true” DMS with a *Ruderman–Kittel–Kasuya–Yosida* (RKKY) mediated ferromagnetic interaction among the TM ions but should rather be considered as an ensemble of superparamagnetic bound polarons, ordering ferromagnetically above the percolation threshold.

## Result and Discussion

Epitaxial films with thicknesses ranging from 15 to 750 nm were deposited by pulsed laser ablation on STO(110) substrates from ZnO and Zn_0.95_Co_0.05_O targets; both targets were also co-doped with 0.2% Al to provide an initial electron doping in order to increase the conductivity of the films. In the following, samples deposited from the ZnO:Al 0.2% and Zn_0.95_Co_0.05_O:Al 0.2% are referred as ZnO and (Zn,Co)O, respectively.

**Figure 1:**
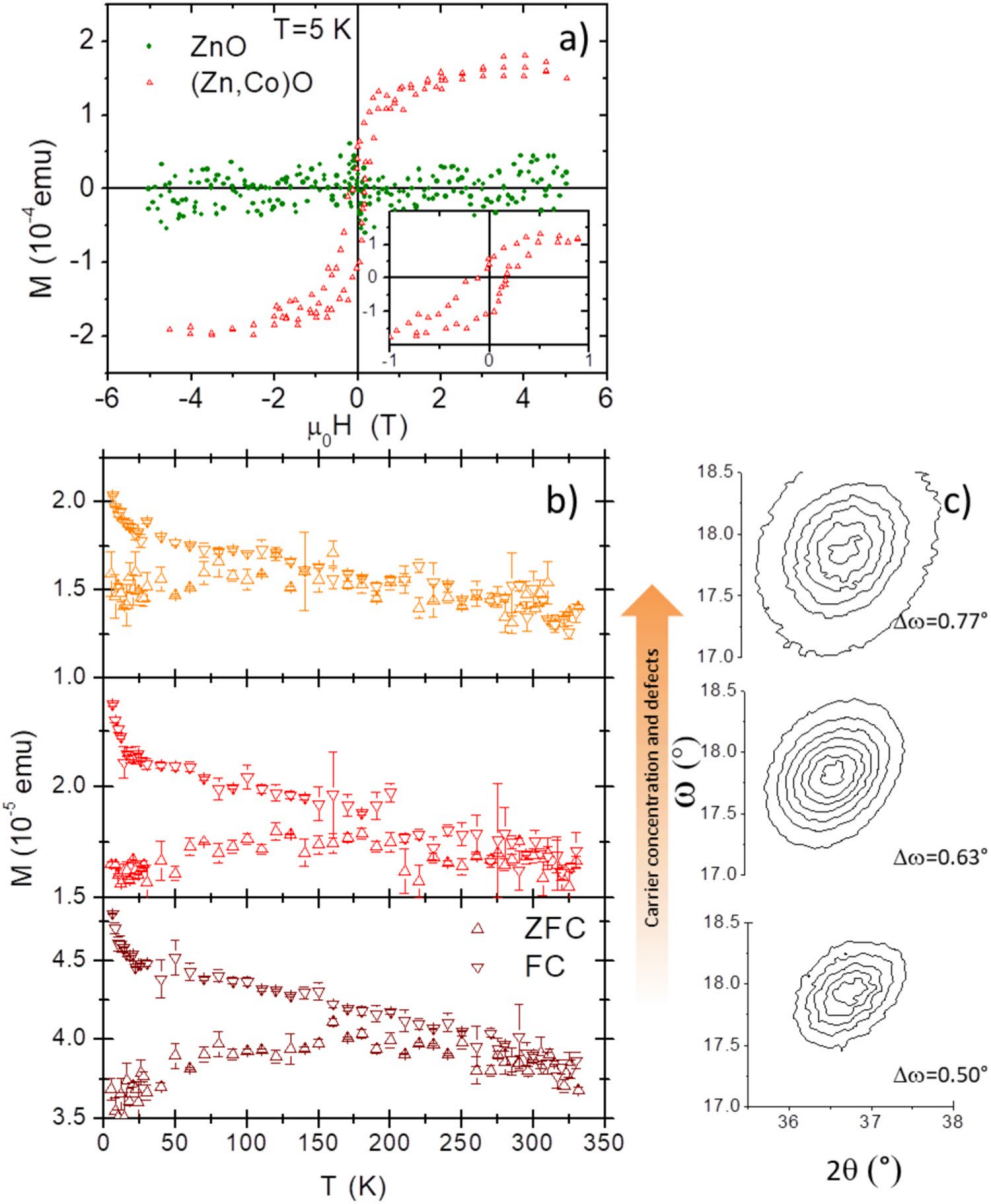
(**a**) Magnetization of ZnO and (Zn,Co)O 700 nm thick films. The data are corrected for the linear diamagnetic contribution of the substrate. In the insert a magnification of the low field region showing hysteresis. (**b**) FC and ZFC magnetization of (Zn,Co)O films prepared under different oxygen pressure ranging from 5 10^−5^ to 810^−3^. The carrier concentration increases from bottom to top panel. (**c**) Reciprocal space map of the corresponding 101 diffraction peak acquired by HRXRD.

The magnetization M as function of the applied magnetic field was measured by SQUID in very thick films (about 700 nm) both for doped and undoped samples. No magnetic signal was detected in ZnO films whereas an “S”-shaped magnetization curve was measured for (Zn,Co)O films up to RT, temperature at which the signal is very small and noisy. The magnetization curve acquired at 5 K is presented in Fig. 1(a). The magnetization of (Zn,Co)O saturates at ≅ 2 T and the saturation magnetization corresponds to ≅ 2 μ_B_ per Co atom. Opening of a small hysteretic circle was observed at low field as highlighted in the figure inset.

**Figure 2:**
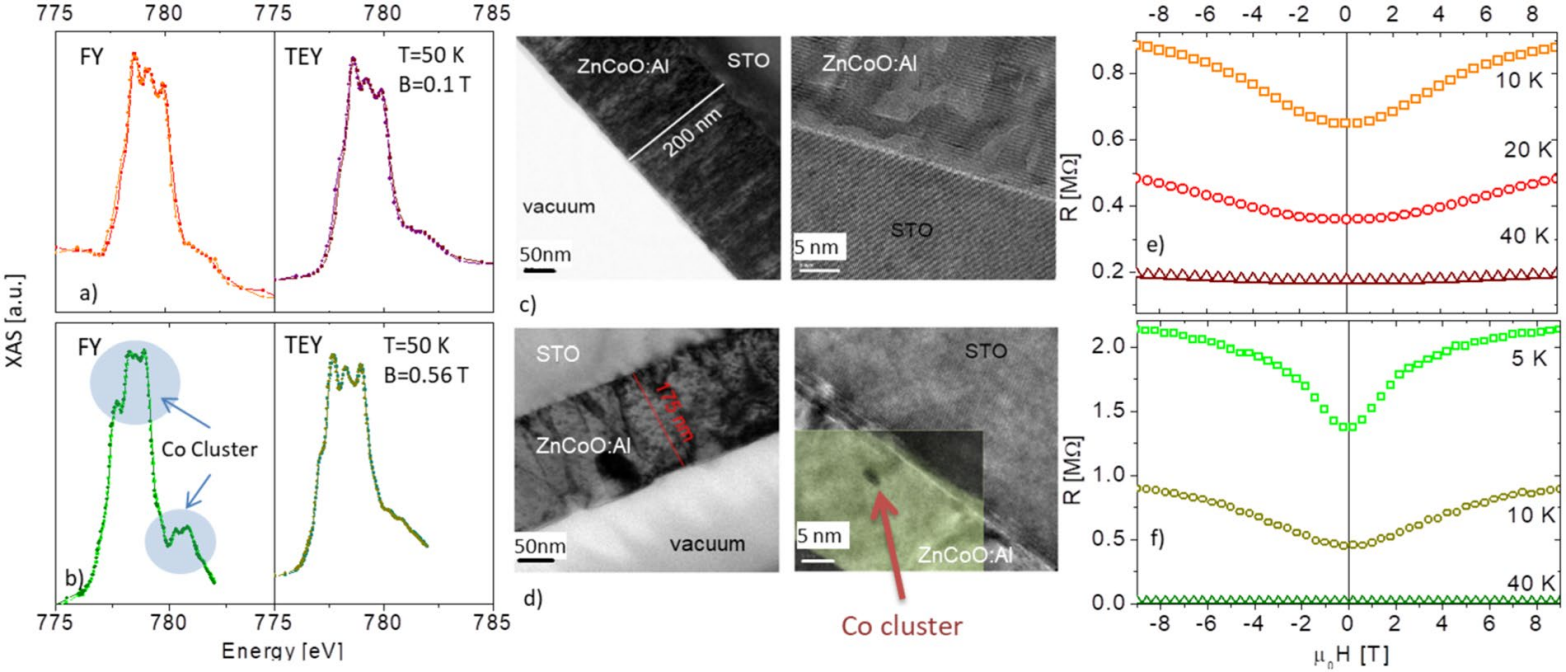
FY and TEY XAS spectra at the L3 Co edge in (Zn,Co)O thin (thickness smaller than 100 nm) films (**a**) without and (**b**) with the presence of Co clusters. No dichroic signal is detected. In panel (c) and (d) HRTEM images of the same samples are reported. In (**d**) the Zn EELS signal (green) is superposed to highlight the presence of a Co cluster. In panel (e and f) the magnetotransport properties of the corresponding samples are reported.

In order to clarify the nature of the magnetism observed in the Co doped samples we measured the field cooled (FC) (cooled in a field of 1T) and zero field cooled (ZFC) magnetization (both measured in a field of 10 mT) of thick films grown under different oxigen pressures in order to modulate carrier concentration and crystal structure (see Fig. 1(b)). Clearly, the temperature at which the ZFC and FC curves separate, which defines the blocking temperature, depends on the growth conditions. It changes from ≈100 K to ≈220 K by increasing the oxygen pressure from 5 10^−5^ to 8 10^−3^ mbar. At the same time, the oxygen pressure also affects the number of defects in the crystalline structure, as demonstrated by the evolution of the HRXRD reciprocal space map of the 101 reflection peak shown in Fig. 2 (c), and the carrier concentration that for the samples shown here are n = 1.2 10^19^; 4.5 10^18^ and 7 10^17^ e^−^/cm^3^,moving from top to bottom. The low temperature splitting of the FC and ZFC curves excludes a simple paramagnetic behaviour of the diluted Co ions. In literature, this separation is often ascribed to the presence of a ferromagnetic ordering; however, the downward curvature of the ZFC curves can be better explained in terms of blocking of superparamagnetic regions^26^. These regions can be nanometric (<5–7 nm) sized Co clusters that are too small to establish a ferromagnetic state^27^ or originate from the formation of bound magnetic polarons (BMP) below the percolation threshold^28^.

X ray spectroscopic adsorption (XAS) can be a powerful tool to investigate the presence of Co cluster^27,29,30^: Opel *et al*.^27^ were able to identify the presence of nanometric Co cluster by the appearance of a secondary peak structure at 781 eV in the Co L_3_ XAS line acquired in Fluorescence yield (FY) mode. We thus performed XAS experiments on (Zn,Co)O films grown at different temperatures obtaining samples with and without the secondary peak in the FY signal (Fig. 2a,b) as also confirmed by High Resolution Transmission Electron Microscopy (HRTEM) analysis (Fig. 2c,d). In particular, the high growth temperature seems to be the main parameter affecting the Co segregation.

**Figure 3:**
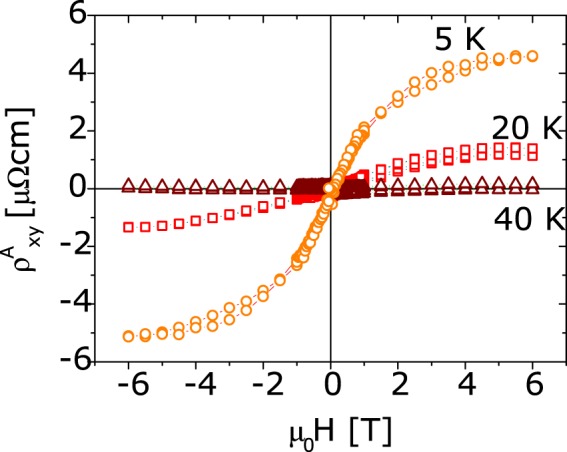
Anomalous Hall Effect in (Zn,Co)O thin films. The linear contribution of the ordinary Hall effect has been subtracted from the experimental data.

We note that both kind of samples (with and without the secondary XAS peak), have similar transport properties, with a large positive magnetoresistance at low temperature (Fig. 2e,f). In fact, both isolated BMP and nanometric sized Co clusters have the same effect on electron transport since they can act as spin scattering centers in magnetoresistance measurements or they can even generate magnetic band spin splitting. The presence of such kind of band splitting is detectable by anomalous Hall Effect measurements^31^ and indeed observed in all our samples, independently on the growth receipe (Fig. 3). We stress that, differently from common expectation, the observation of the anomalous Hall Effect is not a proof of a single phase material (from a magnetic viewpoint)^14^; for example, it was also observed in (Co,Ag) granular systems, which are two immiscible elements (see e.g.^32^.

The previous data clearly demonstrate the difficulty to extract a clear picture of the physics governing the behavior of these systems by comparing samples grown under different conditions, and thus characterized by different degrees of crystallographic order or cluster segregation. A deeper understanding requires the ability to tune the magnetic properties of the very same sample by varying an external parameter, such as electric field, not affecting the sample structural order. In addition, gaining this ability is also crucial in order to realize efficient spintronics devices. For this reason we realized FE transistors having thin film of (Zn,Co)O as active channel and we studied the dependence of their magnetic properties on the free carrier density modulated by FE.

The FE transistors consist of 30–90 nm thick films deposited on STO(110) under the growth conditions excluding clustering as revealed in XAS experiments. The film thickness allows to modulate the charge density in the whole film because is of the same order of magnitude of the Debye screening length ${\lambda }_{D}=\sqrt{\frac{\varepsilon {K}_{B}T}{n{q}^{2}}}$ (∼50 *nm* for ZnO), where ε is the ZnO dielectric constant, *K*_*B*_ the Boltzman constant, *T* the temperature, *n* the unperturbed charge density and *q* the elementary charge.

A detailed characterization of ZnO transport properties under FE can be found in ref.^25^. For sake of clarity, resistivity and carrier density results are summarized here below.

**Figure 4:**
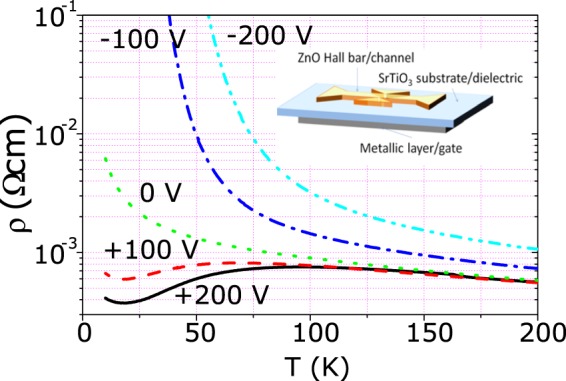
Resistivity of a 80 nm thick ZnO film as a function of temperature at different back-gate voltages. Inset: sketch of the Hall bar shaped Field Effect devices.

Figure 4 shows the resistivity *vs* temperature of an 80 nm thick ZnO film with a carrier density *n* = 5 10^18^ e^−^/cm^3^ for different back-gate voltages. Due to both the 1/*T* behavior of the STO dielectric constant^33^ and the carrier localization inside the ZnO semiconducting channel, the resistivity modulation is dramatically enhanced at low temperatures. In the depletion regime, a marked semiconducting behavior is obtained, whereas, in strong accumulation, metallic-like temperature dependence is observed. The 1/*T* increase of the substrate dielectric constant cannot explain by itself the pronounced metallic trend of the resistance when the temperature decreases. Consequently, the observed metallic behavior probably reflects a real metal-insulator transition of the ZnO channel.

**Figure 5:**
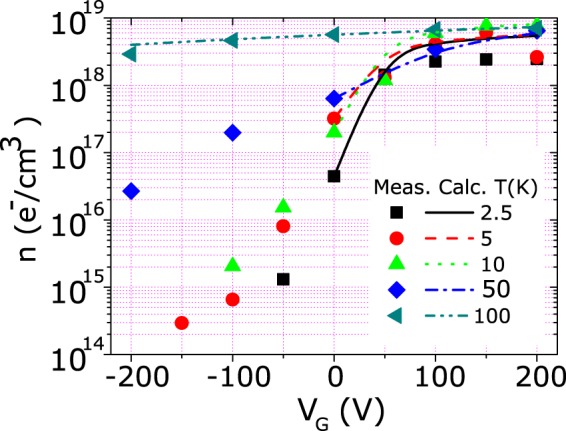
Carrier concentration vs applied Gate voltage for a ZnO FE device measured by the slope of the Hall effect (points) and calculated (lines) from a simple parallel plane plate capacitor model.

In order to elucidate all the details of the FE modulation of transport properties, the temperature and electric field dependence of the STO dielectric properties have to be considered. Assuming a simple parallel plane geometry and adopting the formula of ref.^34^ for the dielectric constant of STO, we evaluated the capacitance of the device *C*, and consequently we estimated the induced charge density as a function of temperature and electric field. Figure 5 shows the measured carrier concentration *n*_*m*_*(V*_*G*_*)*, extracted by Hall Effect measurements at different gate voltages (*V*_*G*_), together with the calculated carrier concentration (${n}_{c}({V}_{G})={n}_{m}(0)+C{V}_{G}$). It can be seen that measured and calculated values are in good agreement in the accumulation region (except for extremely low temperatures, probably because of an overestimation of the STO dielectric constant). On the contrary, in strong depletion, the induced charge density calculated by applying the simple capacitance approach results to be larger than the experimental value and a population inversion should occur. Such an inversion is not experimentally observed possibly due to the presence of a large amount of deep donor levels strongly localized that prevent the Fermi level entering the valence band.

Analogous results were obtained for the magnetic doped samples. These latter showed a similar carrier concentration at zero bias but a higher resistivity, with the films becoming quickly insulating, at negative gate voltages. For this reason, it was possible to span the carrier concentration only from 1.8 10^16^ to 1.1 10^19^ e^−^/cm^3^ (corresponding to a gate voltage ranging from −10 to +200 V). This indicates that a larger disorder is present in the lattice of (Zn,Co)O due to the presence of the magnetic ions.

Due to the small thickness of the films used in FE experiments, the film magnetization can not be reliably measured by SQUID. We therefore preferred to measure the longitudinal magnetoresistance in both ZnO and (Zn,Co)O samples, in a configuration with the magnetic field perpendicular to the film surface and to the current.

**Figure 6:**
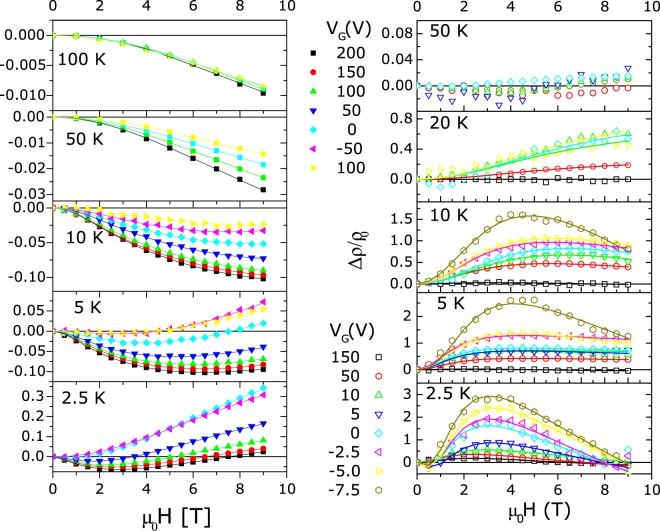
Longitudinal magnetoresistance for both (**a**) ZnO and (**b**) (Zn,Co)O plotted as a function of magnetic field for different applied Gate voltages and temperatures ranging from 2.5K to 100K. Points are the measured data and lines are the best fit according to refs^34,40^ based on the result of Khosla and Fisher^39^.

A very different behaviour of the magnetoresistance (MR) under FE was observed in ZnO and (Zn,Co)O based transistors. The ZnO channel exhibits a large negative magnetoresistance at low field, while, increasing the magnetic field, a positive contribution, bending upward the MR curves, becomes predominant below 10K (Fig. 6(a)). The carrier density modulated by field effect allows to modify the relative weight of these two components and the positive contribution becomes dominant in strong depletion. At higher temperature the same behaviour is observed; however, the negative contribution is predominant for all gate voltages. These results are in agreement with refs^35,36^ where the authors observed an analogous effect in a series of samples with doping ranging from 3.2 × 10^18^ to 1.3 × 10^20^ e^−^/cm^−3^ and 5.5 × 10^18^ to 9.5 × 10^18^ e^−^/cm^−3^, respectively.

In (Zn,Co)O samples a different trend is observed (Fig. 6b). A very large positive MR is observed at low temperature and low magnetic field, when the channel is in high resistivity state (strong depletion), while MR becomes negative in the conductive state (accumulation). These results are in agreement with ref.^37^ where similar magnetotransport properties were observed on a series of Co doped films with carrier concentrations below, close and above the metal to insulator transition. In that paper, the authors stated that the Co doping induces a giant s-d exchange interaction leading to spin splitting of the s-type conduction band. In the metallic regime the spin splitting is small, as well as the magnetic field induced redistribution of electrons between spin split sub-bands; thus, it has a small effect on MR. With decreasing the electron concentration the redistribution increases leading to the positive MR as a consequence of spin-disorder scattering, orbital effects and formation of bound magnetic polarons^38,39^.

A more quantitative analysis of our MR data can be performed taking into account the work of Khosla and Fischer^40^, developed for CdSe, and applied to Zinc Oxide films in refs^34,36,41,42^. According to this approach, the magnetoresistance behaviour can be interpreted taking into account the classical positive MR and a negative term arising from the s-d exchange Hamiltonian.

For carriers in closed orbits the positive term has the form:

1 $$\frac{{\rm{\Delta }}{\rho }_{CO}}{{\rho }_{0}}=\frac{a{B}^{2}}{1+d{B}^{2}}$$

with

2 $$a=\frac{{\sigma }_{1}{\sigma }_{2}{(\mu {}_{1}-\mu {}_{2})}^{2}}{{({\sigma }_{1}+{\sigma }_{2})}^{2}}$$

3 $$d={(\frac{{\sigma }_{1}{\mu }_{1}+{\sigma }_{2}{\mu }_{2}}{{\sigma }_{1}+{\sigma }_{2}})}^{2}$$

where σ_i_ and μ_i_ are the conductivity and mobility of each band and B the magnetic field. Expanding the s-d exchange Hamiltonian to the third order^39^, the negative term can be written as:

4 $$\frac{{\rm{\Delta }}{\rho }_{s-d}}{{\rho }_{0}}=-\,{g}^{2}\,\mathrm{ln}(1+{h}^{2}{B}^{2})$$

with

5 $${g}^{2}={A}_{1}J{\rho }_{F}[S(S+1)+ < \,{M}^{2} > ]$$

6 $${h}^{2}=[1+4{S}^{2}{\pi }^{2}{\frac{(2J{\rho }_{F})}{{g}_{0}}}^{4}]\cdot {(\frac{{g}_{0}\mu }{\alpha KT})}^{2}$$

where *J* is the exchange Integral, ρ_*F*_ the density of states at the Fermi energy, *g*_0_ the gyromagnetic factor, <*M>* the average magnetization, *S* the spin of the localized magnetic moment, and α is a numerical constant. *A*_1_ is considered to be a measure of the spin based scattering.

**Figure 7:**
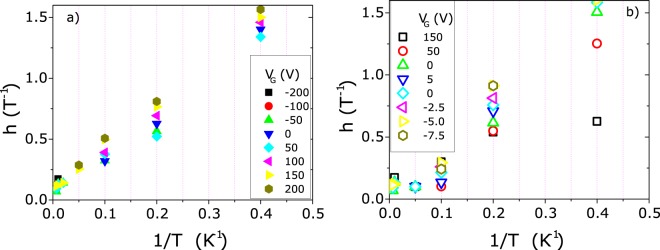
*h* fitting parameter versus 1/T for (**a**) ZnO and (**b**) (Zn,Co)O. The expected linear behavior indicates the robustness of the fit.

The magnetoresistance data of both samples can be fitted (solid lines in Fig. 6) with the two above cited contributions using *a,d, g* and *h* as fitting parameters. The agreement of the least-squares fits to the data is excellent for both samples at all gate voltages and temperatures. The fitting parameters show a systematic and smooth carrier concentration and temperature dependence. As a test of the robustness of the fit we plotted in Fig. 7a and b the *h* fitting parameter as a function of 1/T for both ZnO and (Zn,Co)O. As expected from eq. 5, a linear behavior is observed. The slope of the fitting line, determined by the other unknown coefficient in eq. 5, monotonically increases with the carrier concentration in ZnO, whereas the opposite behavior is observed in (Zn,Co)O. *h* values and slopes are similar in both cases and the change of the slope with the carrier concentration is small. This suggest that the parameters determining the *h* vs 1/T behavior are similar for the two samples and slightly dependent on the carrier density.

**Figure 8:**
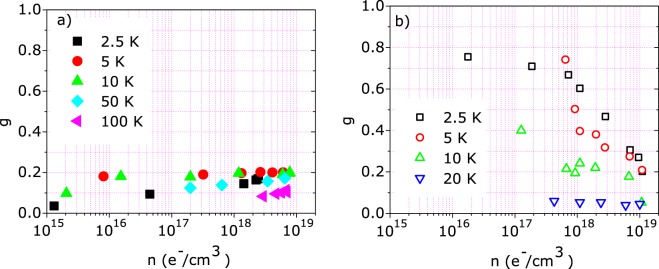
*g* fitting parameter as a function of the carrier concentration for (**a**) ZnO and (**b**) (Zn,Co)O. The carrier concentration was inferred from the Hall Effect measurements.

Figure 8 shows the g fitting parameter as a function of the measured carried concentration. As highlighted in eq. 6, g monotonically depends on the sample magnetization and the same parameters determining the temperature dependence of h. Since such parameters are weakly dependent on the carrier concentration and have similar values in both ZnO and (Zn,Co)O, we can, in first approximation, consider *g* as representative of the sample magnetization. In the case of ZnO (Fig. 8a), *g* is small and rather independent on the carrier concentration with values ranging from 0.1 to 0.2 as the temperature is varied from 100 K to 2.5 K. A very different behavior is observed for (Zn,Co)O (Fig. 8b): *g* is larger (about 0.8) at low temperature and low carrier concentration, strongly decreasing to about 0.1 when carrier concentration or temperature are increased to 10^19^ e^−^/cm^3^ or 20 K, respectively.

Taking *g* as a measure of the magnetization, we infer that in (Zn,Co)O the magnetization is controlled by the carrier density: this effect is peculiar of a RKKY or BMP model and it is not explained by the mere presence of isolated cobalt cluster. Moreover, since the carrier concentration is modulated by FE, it is possible to control the magnetization of (Zn,Co)O by the electric field.

**Figure 9:**
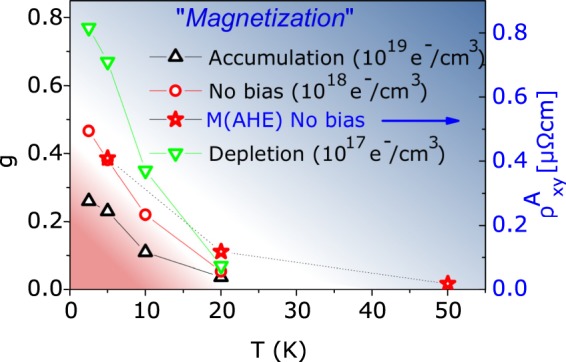
Estimation of the (Zn,Co)O sample magnetization from the g fitting parameter and the anomalous Hall effect. The left and right vertical scale are chosen in such a way that the point at 5K and zero bias coincide.

The evolution of the magnetization with temperature can also be inferred from the *g* values. In Fig. 9, *g* data corresponding to accumulation, zero bias and depletion regimes are plotted as a function of the temperature. For comparison, in the same figure we also show the saturation magnetization obtained from the anomalous Hall Effect measured at zero bias (Fig. 3). The two behaviors at zero bias are in fair agreement and a magnetic ordering temperature between 20 and 50 K can be inferred for this particular sample. When charge is accumulated or depleted the magnetization is suppressed or enhanced respectively whereas no relevant effects on the ordering temperature are visible.

**Figure 10:**
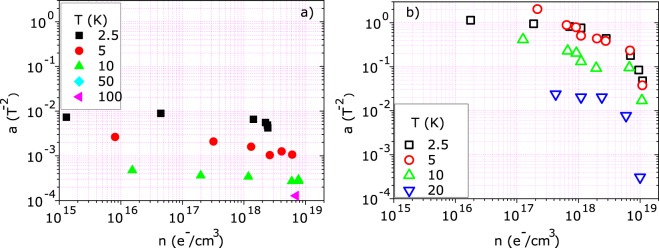
*a* fitting parameter as a function of the carrier concentration for (**a**) ZnO and (**b**) (Zn,Co)O. The carrier concentration was inferred from the Hall Effect.

An estimation of the positive magnetoresistance is given by the *a* fitting parameter whose dependence on the carrier concentration for ZnO and (Zn,Co)O is plotted in Fig. 10. The behavior of the two samples is very different, as observed for the *g* parameter, indicating that different scattering and exchange mechanisms are involved when magnetic ions are present.

According to refs^35,43^, on the basis of the study reported in^44^, the giant positive magnetoresistance can be attributed to the magnetic scattering of spin polarized charge carriers. In those papers a direct relationship between the giant MR values and the spin polarization P is established. The authors estimated in about 1 meV the spin splitting and found a decrease of P with increasing the temperature and the number of free carriers.

**Figure 11:**
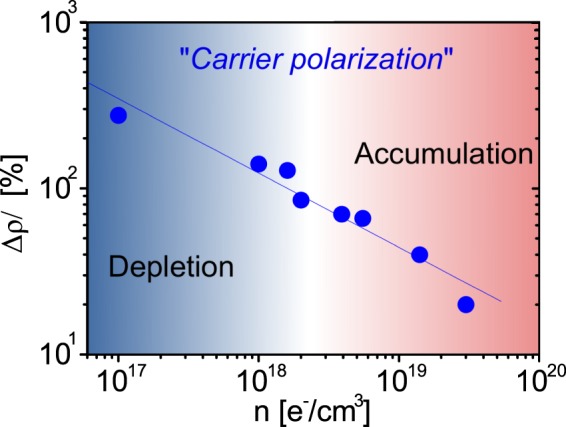
Maximum positive value of the low temperature magnetoresistance in (Zn,Co)O as a function of the carrier concentration inferred from Hall Effect. According to ref  ^35,42,43^ it is directly related to the spin carrier polarization.

Consistent results are obtained applying their model to our data. The giant positive MR, plotted in Fig. 11, decreases with increasing the carrier concentration. Consequently, by applying the above cited model, also the spin carrier polarization decreases with increasing carrier concentration and temperature and vanishes above 10^19^ e^−^/cm^3^ and 20 K, respectively, in agreement with other works^36^. The ability to induce by field effect such large modulation of the MR and, consequently, of the spin polarization is a fundamental step towards spintronics applications.

## Result Interpretation

All our results can be well explained in term of magnetic ordered regions governed by the bound magnetic polaron percolation theory. This model, originally proposed for oxides by Coey *et al*.^28^, is based on the formation of a spin polarized cloud around a region containing both a donor defect and a magnetic impurity. When number or size of these clouds increases, the BMP can overlap and form magnetically ordered regions. In turn, these regions can overlap and pervade the whole sample producing a situation analogous to a ferromagnetic state.

In particular three articles are relevant for our work: in^16^ Chou *et al*. studied the dependence of the magnetic properties on carrier concentration modulated by FE. Their data were in agreement with a modified BPM model but only a modulation of a few percent was achieved. In Behan *et al*.^4^ several samples with carrier concentration ranging from 10^17^ to 10^21^ e^−^/cm^3^ were analyzed, finding an agreement with the BPM model at low doping and with a direct carrier mediated ferromagnetism in the very high doping regime. A similar result was obtained by Lu *et al*.^7^ in (Zn,Co)O co-doped with Ga for high doping levels. The main role of defects in magnetism propagation was also recently re-proposed by Andriotis and Menon^45^ and Qi *et al*.^46^ in a theoretical and experimental work, respectively.

**Figure 12:**
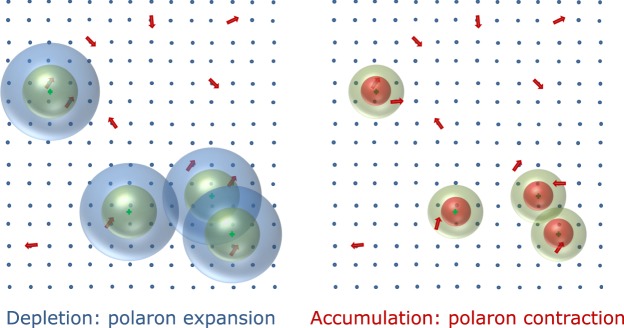
Schematic drawings for the size evolution of bounded magnetic polaron with carrier concentration. Red arrows and cyan dots represent magnetic impurities and donor defects, respectively; blue, red and green spheres represent the expanded, contracted and natural polaron sizes, respectively. The increase in carrier density reduces the polaron radius and consequently increases the BMP separation; thus, the magnetic coupling decreases (right). The opposite situation happens when the carrier density decreases (left).

In our experiment, number and position of the BMP is constant since we are not varying the number of donor and magnetic defects; however, by acting on the free carrier concentration, we modulate the volume on which these defects are active, i.e. we modulate the BMP radius. In addition, by increasing the distance on which the donor defect is active, new polarons can form when a magnetic impurity is included in its effective sphere. Following the above cited references, the radius of a polaron is ${r}_{H}=\varepsilon (\frac{m}{{m}^{\ast }}){a}_{0}$ where ε is the high frequency dielectric constant, *m* the electron mass, m* the effective mass and *a*_0_ the Bohr radius. The increase of the carrier concentration induced by the electric field increases the effective mass of bounded carriers. This effect of the non-parabolic conduction band is well established in many transparent conducting oxides^47^ in general and in Zinc oxide thin films in particular^48–50^. The increase in the effective mass reduces the polaron radius leading to an increased BMP separation and, consequently, to a decrease of the magnetic coupling, as sketched in Fig. 12.

To summarize, the carrier density *n* modulated by FE, determining polaron size and number, establishes the threshold for the polaron percolation and thus for the formation of long range ferromagnetic order. From the *g* dependence on *n* shown in Fig. 8 we can affirm that the polaron percolation is completed for *n* < 10^18^ e^−^/cm^3^. At the same time, the polaron size affects the carrier spin polarization. As shown by Fig. 11, *P* increases monotonically with decreasing *n*, and it seems to be not affected by the ferromagnetic order formation. We stress that *P* changes by more than one order of magnitude for the range of carrier density considered here. The ability to control such large variation of the carrier spin polarization is crucial for possible spintronics applications.

The magnetic properties of (Zn,Co)O are thus the result of a very subtle interplay between various kind of defects (pure donor, pure magnetic ions, or defect with both properties like Oxygen vacancies), the microstructure (i.e. the presence of magnetic clusters) and the free electron density. In this context, it is not surprising that results are strongly sample dependent and different properties have been reported by several authors in the last years. Field effect is a powerful technique allowing to study the effect of one of the main parameter, i.e. the charge density, on the same sample without altering any other condition.

Also the apparently contradictory results reported by both Behan *et al*. (ref.^4^) and Lu *et al*. (ref.^7^), who found an increased magnetization at very high carrier concentration can be explained. Indeed, samples in the above cited articles are heavily doped to reach a nearly metallic regime. This implies a very high amount of defects that can strongly modify the polaron density (and possibly introduce different impurity based coupling mechanism), and allow percolation of the magnetization throughout the whole sample despite their very small size due to the high carrier density. On the contrary in our work the carrier concentration is enhanced by Field Effect thus keeping untouched the high structural order and ruling out the role of defects.

The disappearing of magnetic order at large carrier concentration can also explain the lack of reliable magnetic signal at remanence in XMCD measurements as reported by our group^51^ and other authors^52,53^. The exposure of the sample to a high brilliant x-ray beam produces a large amount of photoelectrons, that, analogously to the FE induced carriers, drops the resistivity (during our XAS experiment a reduction of ρ by about two orders of magnitude was measured) and shrinks down the polaron size, thus destroying the possibly preexistent magnetic order. We note that recently, it has also been reported that this large amount of photoelectrons is responsible for the reduction of the spin lifetime in single ion molecular magnets and single atom magnets at surface^54–57^.

## Conclusions

In conclusion, magnetotransport properties of pure and cobalt doped zinc oxide field effect transistors were studied. By adopting the Khosla and Fisher model the transport properties were used to evaluate the spin carrier polarization and sample magnetization as a function of carrier concentration. It was found that increasing the free carrier concentration, both spin carrier polarization and magnetization decrease. This result was interpreted in the framework of a BPM model in which the carrier concentration modifies the polaron size and, to a lesser extent, their number.

Our results strengthen the hypothesis that TM doped ZnO is not a “true” DMS with a RKKY mediated ferromagnetic interaction among the TM ions but should rather be considered as an ensemble of superparamagnetic bound polarons, ordering ferromagnetically above the BMP percolation threshold. However, the possibility of a large modulation, by electric field, of the spin carrier polarization is of great relevance for spintronics applications irrespective of homogeneus or inhomogeneus status of the material.

## Method

Epitaxial films with thicknesses ranging from 15 to 750 nm were deposited by pulsed laser ablation (λ = 248 nm and fluency ≈ 2 J/cm^3^) on 110 oriented STO substrates from a sintered ZnO, and Zn_0.95_Co_0.05_O, targets; both targets are also co-doped with 0.2% Al to provide an initial electron doping in order to increase the conductivity of the films. The pellets were prepared by solid state reaction of stoichiometric oxides mixture at 1000 °C for 24 hours. The deposition was carried out in two steps: at first, a 2 nm thick ZnO relaxation layer was grown at 600 °C; then the temperature was raised up to 750 °C and a layer with high crystalline quality was deposited. The thickness of the relaxation layer was chosen as the minimum thickness necessary to obtain a uniform flat coverage of the substrate surface as evaluated by *in-situ* Reflection High Energy Electron Diffraction (RHEED) analysis. Details about films deposition and full structural characterization are reported in refs^21,25^.

A great care to avoid any magnetic contamination of the samples was used, in particular film for magnetic characterization were never manipulated with metal tools.

Field Effect (FE) devices were realized using the substrate as dielectric and realizing the gate contact by silver paste on the back side of the substrate (back Gate geometry). The films were patterned by photolithography and wet etching in order to obtain a Hall-bar-shaped channel. Complete description of FE devices are reported elsewhere^23,25^; here we focus on the use of these devices for the study of the carrier effect on transport and magnetic phenomena. In order to induce a large amount of carriers thin films (50–80 nm) were employed for the FE devices.

Standard XRD, SEM and AFM analysis were performed to check the overall quality of the Zinc oxides layer. In order to exclude the presence of Co precipitates we perform XAS and XPEEM measurements at the SIM beam line of the Swiss Light Source (SLS). TEM analysis was performed at EPFL, Lausanne; full result about these measurements are reported in ref.^51^.

Magnetic measurements were performed by a Quantum Design DC magnetometer SQUID from 5 to 330 K at magnetic field up to 5 T. Magnetic measurements were carried out on the thickest films (750 nm) in order to maximize the signal from the layer, and on blank substrates of the same batch in order to subtract the diamagnetic contribution of SrTiO_3_.

Resistivity, magnetoresistivity and Hall Effect under FE were measured in a Quantum Design PPMS from 2.5 to 330 K and in magnetic field up to 9T. Four wire measurements were done with external instruments Keithley 192 nanovoltmeters and Keithley 110 current source. In case of very strong depletion and very high channel resistance, a two wire configuration with a Keithley 6487 picoammeters was employed. The system allows for measurements with gate polarization up to 250 V (+−) without any detectable leakage. Gate voltage and current were controlled by a Keithley 6487 picoammeters.

## Data Availability

The authors claim that all the supporting data is available from the authors.

## References

[1] Dietl T, Ohno H, Matsukura F, Cibert J, Ferrand D (2000). Zener model description of ferromagnetism in zinc-blende magnetic semiconductors. Science.

[2] Jung SW (2002). Ferromagnetic properties of Zn_1−x_Mn_x_OZnO epitaxial thin films. Appl. Phys. Lett..

[3] Liu XC (2006). Magnetic properties of nn-type Cu-doped ZnO thin films. Appl. Phys. Lett..

[4] Behan AJ (2008). Two magnetic regimes in doped ZnO corresponding to a dilute magnetic semiconductor and a dilute magnetic insulator. Phys. Rev. Lett..

[5] Kittisveld KR, Liu WK, Gamelin DR (2006). Electronic structure origins of polarity-dependent high-T_C_ ferromagnetismin oxide-diluted magnetic semiconductors. Nat. Material.

[6] Liu Y, MacManus-Driscoll JL (2009). Impurity control in Co-doped ZnO films through modifying cooling atmosphere. Appl. Phys. Lett..

[7] Lu Z (2009). The origins of ferromagnetism in Co-doped ZnO single crystalline films: From bound magnetic polaron to free carrier-mediated exchange interaction. Appl. Phys. Lett..

[8] Fukumura T (2001). Magnetic properties of Mn-doped ZnO. Appl. Phys. Lett..

[9] Yin S (2006). Absence of ferromagnetism in bulk polycrystalline Zn_0.9_Co_0.1_O. Phys. Rev. B.

[10] Ueda K, Tabata H, Kawai T (2001). Magnetic and electric properties of transition-metal-doped ZnO films. APL.

[11] Ne A (2008). Absence of intrinsic ferromagnetic interactions of isolated and paired co dopant atoms in Zn_1−x_Co_x_O with high structural perfection. Phys.Rev. Lett..

[12] Ney A (2010). Advanced spectroscopic synchrotron techniques to unravel the intrinsic properties of dilute magnetic oxides: the case of Co:ZnO. New Journal of physics.

[13] Xu XH (2006). Carrier-induced ferromagnetism in n-type ZnMnAlO and ZnCoAlO thin films at room temperature. New Journal of Physics.

[14] Ogale, S. B. Dilute doping, defects, and ferromagnetism in metal oxide systems. *Adv*. *Mat* **22**, 3125–3155 (2010). 10.1002/adma.200903891 20535732

[15] Yang, Z. A perspective of recent progress in ZnO diluted magnetic semiconductors. *Appl Phys A* **112**, 241–254 (2013).

[16] Chou H, Lin CP, Huang JCA, Hsu H (2008). Magnetic coupling and electric conduction in oxide diluted magnetic semiconductors. Phys. Rev. B.

[17] Lee HJ, Helgren E, Hellman F (2009). Gate-controlled magnetic properties of the magnetic semiconductor (Zn,Co)O. Appl. Phys. Lett..

[18] Chang L (2014). Electric-Field control of ferromagnetism in Mn-doped ZnO nanowires. NanoLett..

[19] Wang XL, Shao Q, Leung CW, Lortz R, Ruotolo A (2014). Non-volatile, electric control of magnetism in Mn-substituted ZnO. Appl. Phys. Lett..

[20] Xie J (2017). Light control of ferromagnetism in ZnO films on Pt substrate at room temperature. Sci. Rep..

[21] Bellingeri E, Marré D, Pallecchi I, Pellegrino L, Siri AS (2005). High mobility in ZnO thin films deposited on perovskite substrates with a low temperature nucleation layer. Appl. Phys. Lett..

[22] Bellingeri E, Marré D, Pallecchi I, Pellegrino L, Siri AS (2005). Deposition of ZnO thin films on SrTiO3 single-crystal substrates and field effect experiments. Thin Solid Films.

[23] Bellingeri E (2005). High mobility ZnO thin film deposition on SrTiO3 and transparent field effect transistor fabrication. Superlattices And Microstructures.

[24] Bellingeri E (2008). Crystalline ZnO/SrTiO_3_ transparent field effect transistor. D Physica Status Solidi A-Applications And Materials Science.

[25] Bellingeri E (2007). Transport properties of non magnetic and magnetic ZnO thin films under field effect. Proc. SPIE.

[26] Quan Z-Y, Zhang L, Liu W, Zeng H, Xu X-H (2014). Resistivity dependence of magnetoresistance in Co/ZnO films. Nanoscale Research Letters.

[27] Opel M (2008). Nanosized superparamagnetic precipitates in cobalt-doped ZnO, Eur. Phys J. B.

[28] Coey JMD, Venkatesan M, Fitzgerald CB (2005). Donor impurity band exchange in dilute ferromagnetic oxides. Nature Mater..

[29] Zhang Y, Wang Z, Cao X (2013). Characterization of Co distribution in ZnO by x-ray magnetic circular dichroism. Journal of Applied Physics.

[30] Seo S-Y (2013). X-ray absorption fine structure study of cobalt ion distribution in ferromagnetic Zn_1−x_Co_x_O films. J. Phys.: Condens. Matter.

[31] Higgins JS, Shinde SR, Ogale SB, Venkatesan T, Greene RL (2004). Hall effect in cobalt-doped TiO_2-δ_. Physical Review B.

[32] Xiong P (1992). Extraordinary Hall effect and giant magnetoresistance in the granular Co-Ag system. Phys. Rev. Lett..

[33] Sakudo T, Unoki H (1971). Dielectric properties of SrTiO_3_ at low temperatures. Phys Rev. Lett..

[34] Neville RC, Hoeneisen B, Mead CA (1972). Permittivity of strontium titanate. J. Appl. Phys..

[35] Reuss F (2005). Magnetoresistance in epitaxially grown degenerate ZnO thin films. Appl. Phys. Lett..

[36] Ghoshal, S. & Kumar, P. Magneto-transport study of pure and Co doped ZnO thin films. IEEE Transactions on Magnetics, **48**, NO. 11, (2012).

[37] Xu Q (2006). Metal-insulator transition in Co-doped ZnO: Magnetotransport properties. Phys. Rev. B.

[38] Dietl, T. In Semi magnetic semiconductors and diluted magnetic semiconductors (eds Averous, M. & Balkanski, M.) (Plenum Press, New York, 1991).

[39] Lee PA, Ramakrishnan TV (1985). Disordered electronic systems. Rev. Mod. Phys..

[40] Khosla RP, Fischer JR (1970). Magnetoresistance in degenerate CdS: Localized magnetic moments. Phys. Rev. B.

[41] Gacic M (2007). Magnetism of Co-doped ZnO thin films. Phys. Rev. B.

[42] Modepalli V (2016). Gate-tunable spin exchange interactions and inversion of magnetoresistance in single ferromagnetic ZnO nanowires. ACS Nano.

[43] Xu Q (2007). Magnetoresistance and anomalous Hall effect in magnetic ZnO films. J. Appl. Phys..

[44] Andrearczyk T (2005). Spin-related magnetoresistance of n-type ZnO:Al and Zn1−xMnxO:Al thin films. Phys. Rev. B.

[45] Andriotis AN, Menon M (2013). Defect-induced magnetism: Codoping and a prescription for enhanced magnetism. Phys. Rev B.

[46] Qi S (2011). Carrier-mediated nonlocal ferromagnetic coupling between local magnetic polarons in Fe-doped In2O3 and Co-doped ZnO. Phys. Rev. B.

[47] Pisarkiewicz T, Zakrzewska K, Leja E (1989). Scattering of charge carriers in transparent and conducting thin oxide films with a non-parabolic conduction band. Thin Solid Films.

[48] Abdolahzadeh A, Ziabari S, Rozati M (2012). Carrier transport and bandgap shift in n-type degenerate ZnO thin films: The effect of band edge nonparabolicity. Physica B: Condensed Matter Volume.

[49] Young DL, Coutts TJ, Kaydanov VI, Gilmore AS, Mulligan WP (2000). Direct measurement of density-of-states effective mass and scattering parameter in transparent conducting oxides using second-order transport phenomena. J. of Vacuum Science & Technology A.

[50] Tang J (2014). Determination of carrier concentration dependent electron effective mass and scattering time of n-ZnO thin film by terahertz time domain spectroscopy. Journal of Applied Physics.

[51] Bellingeri E (2008). Field effect controlled ferromagnetism in transition metal doped ZnO. Proc of SPIE.

[52] Barla A (2007). Paramagnetism of the Co sublattice in ferromagnetic Zn_1−x_Co_x_O films. Phys. Rev. B.

[53] Tietze T (2008). XMCD studies on Co and Li doped ZnO magnetic semiconductors. New J. Phys..

[54] Dreiser J (2014). X-ray Induced Demagnetization of Single-Molecule Magnets. Appl. Phys. Lett..

[55] Wäckerlin C (2016). Giant Hysteresis of Single-Molecule Magnets Adsorbed on a Nonmagnetic Insulator. Adv. Mater..

[56] Donati F (2016). Magnetic remanence in single atoms. Science.

[57] Baltic R (2016). Superlattice of Single Atom Magnets on Graphene. Nano Lett..

